# Myopia Prediction Using Machine Learning: An External Validation Study

**DOI:** 10.3390/vision9040084

**Published:** 2025-10-09

**Authors:** Rajat S. Chandra, Bole Ying, Jianyong Wang, Hongguang Cui, Guishuang Ying, Julius T. Oatts

**Affiliations:** 1 Perelman School of Medicine, University of Pennsylvania, Philadelphia, PA 19104, USA; rajatsc4@gmail.com; 2 School of Arts and Sciences, University of Pennsylvania, Philadelphia, PA 19104, USA; boleying1031@gmail.com; 3 Department of Ophthalmology, The First Affiliated Hospital, College of Medicine, Zhejiang University, Hangzhou 310023, China; wangjy4@zju.edu.cn (J.W.); 1189002@zju.edu.cn (H.C.); 4 Department of Ophthalmology, Perelman School of Medicine, University of Pennsylvania; Philadelphia, PA 19104, USA; 5 Division of Ophthalmology, Children’s Hospital of Philadelphia, Philadelphia, PA 19104, USA

**Keywords:** validation, machine learning, myopia, prediction, cycloplegic refraction

## Abstract

We previously developed machine learning (ML) models for predicting cycloplegic spherical equivalent refraction (SER) and myopia using non-cycloplegic data and following a standardized protocol (cycloplegia with 0.5% tropicamide and biometry using NIDEK A-scan), but the models’ performance may not be generalizable to other settings. This study evaluated the performance of ML models in an independent cohort using a different cycloplegic agent and biometer. Chinese students (N = 614) aged 8–13 years underwent autorefraction before and after cycloplegia with 0.5% tropicamide (*n* = 505) or 1% cyclopentolate (*n* = 109). Biometric measures were obtained using an IOLMaster 700 (*n* = 207) or Optical Biometer SW-9000 (*n* = 407). ML models were evaluated using R^2^, mean absolute error (MAE), sensitivity, specificity, and area under the ROC curve (AUC). The XGBoost model predicted cycloplegic SER very well (R^2^ = 0.95, MAE (SD) = 0.32 (0.30) D). Both ML models predicted myopia well (random forest: AUC 0.99, sensitivity 93.7%, specificity 96.4%; XGBoost: sensitivity 90.1%, specificity 96.8%) and accurately predicted the myopia rate (observed 62.9%; random forest: 60.6%; XGBoost: 58.8%) despite heterogeneous cycloplegia and biometry factors. In this independent cohort of students, XGBoost and random forest performed very well for predicting cycloplegic SER and myopia status using non-cycloplegic data. This external validation study demonstrated that ML may provide a useful tool for estimating cycloplegic SER and myopia prevalence with heterogeneous clinical parameters, and study in additional populations is warranted.

## 1. Background

Myopia is a growing global public health concern predicted to affect 4.8 billion people by 2050 [1]. Early detection for timely intervention to prevent myopia-associated vision-threatening conditions is key. Additionally, close monitoring of myopia prevalence, severity, and progression to high myopia is warranted at both the individual and population levels to reduce the global burden of myopia. Measuring refractive error following cycloplegia is the gold standard for detecting myopia and quantifying myopia severity [2].

Obtaining cycloplegic refractive error in population-based epidemiology studies requires significant time and resources, which can limit study feasibility or the accuracy of myopia prevalence data [3,4,5,6,7]. Using non-cycloplegic refractive error leads to overestimation of myopia prevalence and severity, which biases study results [7,8]. To improve myopia detection, severity classification, and risk factor determination under non-cycloplegic conditions, different strategies have been evaluated, including both traditional statistical prediction models and modern machine learning models [9,10,11,12,13,14,15,16,17,18,19,20].

Among the previously described models for predicting cycloplegic refractive error using non-cycloplegic data, machine learning models have shown the greatest promise [17,18]. Recently, we trained six ML models to predict cycloplegic spherical equivalent refraction (SER) and myopia status using easily obtainable measures under non-cycloplegic conditions in 1938 students aged 5 to 18 years from one rural city (Jinyun) and validated the models in 1476 students in an urban city (Hangzhou), enrolled during October 2020 to January 2021 [18]. The six ML models were support vector machine (SVM), random forest (RF), extreme gradient boosting (XGBoost), multi-layer perceptron (MLP) neural network, linear regression, and lasso regression. Among these six ML models, XGBoost performed best for predicting cycloplegic SER, and RF performed best for predicting myopia status with high sensitivity and specificity [18]. However, the measures taken from these students for training and validation followed the same protocol (e.g., 0.5% tropicamide for cycloplegia, NIDEK A-scan biometry). As a result, the high predictive performance observed may not generalize well to settings where different cycloplegic agents or ocular biometers are used. To address this limitation, we conducted an independent validation study involving students aged 8 to 13 years in Hangzhou between December 2023 to January 2024. In this study, cycloplegic refraction was performed using either 0.5% tropicamide or 1% cyclopentolate, and ocular biometry was measured using either the IOL Master 700 or the SW-9000 device. The goal of this validation study was to assess the performance of the two best-performing ML models from our prior work —XGBoost (for predicting cycloplegic SER) and random forest (for predicting myopia status)—in an independent cohort under varied clinical protocols.

## 2. Methods

From December 2023 to January 2024, students were enrolled from an elementary school (n = 267 students in grades 3 or 4) and a middle school (n = 347 students in grade 7) in Hangzhou. Human subject research approval was obtained from Zhejiang University and the local Administration of the Education and School Board (Protocol Approval Number: IIT20210265A). Written informed consent was obtained from each participant’s parent or legal guardian. The study followed the tenets of the Declaration of Helsinki.

### 2.1. Eye Examinations and Ocular Biometry

All students underwent a comprehensive eye examination by an ophthalmologist. This included uncorrected visual acuity (UCVA) testing with a retro-illuminated logMAR chart with tumbling-E optotypes and ocular biometry using an IOLMaster 700 (Carl Zeiss Meditec AG, Jena, Germany) or Optical Biometer SW-9000 (Tianjin SuoWei Electronic Technology, Tianjin, China) under non-cycloplegic conditions. Recorded biometric parameters included axial length (AL), corneal curvature radius (CR), central corneal thickness, and anterior chamber depth. Students also underwent autorefraction before and after cycloplegia with 0.5% tropicamide (n = 505) or 1% cyclopentolate (n = 109) using a table-mounted NIDEK autorefractor (Model ARK-510A, Gamagori, Japan). For cycloplegic autorefraction, one drop of 0.5% tropicamide or 1% cyclopentolate was instilled in each eye every 5 min for 4 administrations, and cycloplegic refractive error was taken 30 min after the last administration. For both non-cycloplegic and cycloplegic refractive errors, three readings were taken from each eye. If the difference between any of two readings of sphere or cylinder from an eye was greater than 0.5 diopters (D), the refractive error measurement was repeated. For each eye, the average of the three readings of refractive error was used for statistical analysis.

### 2.2. Machine Learning Models

We previously developed and validated six ML models for predicting cycloplegic SER and myopia status using contemporaneous non-cycloplegic measures [18]. In the validation data, we found that XGBoost performed best for predicting cycloplegic SER, and random forest performed best for predicting myopia status. This study evaluated these two best-performing ML models for validation. We applied ML models for predicting two outcome measures of cycloplegic refractive error in each eye including (1) cycloplegic spherical equivalent refraction (SER), calculated as sphere + 0.5 * cylinder; (2) myopia, defined as cycloplegic SER ≤ −0.5 diopter (D) considering the negative sign (i.e., −0.5 D or worse). The predictors for the ML models were as follows: demographics (age, gender), wearing refractive correction (yes/no), eye laterality (left eye, right eye), non-cycloplegic SER, biometric measures (AL, CR, AL/CR ratio, central corneal thickness, and anterior chamber depth), intraocular pressure, and UCVA (in logMAR).

The random forest model makes predictions by combining the results of a collection of tree predictors while avoiding overfitting even with the addition of more trees [21]. The XGBoost model is similar to the random forest model in that it uses an ensemble of tree predictors, but it also uses gradient tree boosting and is trained in an additive manner [22]. The performance of XGBoost was evaluated using R^2^ and mean absolute error (MAE) for predicting the cycloplegic SER of an eye and for predicting myopia using sensitivity and specificity (with myopia defined as predicted cycloplegic SER ≤ −0.5 D). The performance of the random forest model for predicting myopia status was evaluated using accuracy, the area under the receiver operating characteristic (ROC) curve (AUC), sensitivity, and specificity. To quantify the importance of each feature used in the predictions, we used the permutation importance, defined by the decrease in the model’s R^2^ for predicting cycloplegic SER and the decrease in the model’s AUC for predicting myopia status after shuffling each feature [21].

We used Python 3.9 and its open-source package scikit-learn version 1.1.3 to implement these ML models [23]. The code for our ML analysis can be provided upon reasonable request to the authors. Using the predicted cycloplegic SER for each eye from the XGBoost model, we further evaluated model performance by calculating the following: (1) the mean relative difference and mean absolute difference between predicted and observed cycloplegic SER overall, by age group, by level of cycloplegic SER, by type of cycloplegic agent, and by biometer; (2) the Pearson correlation coefficient between predicted cycloplegic SER and observed cycloplegic SER by age group, by type of cycloplegic agent, and by biometer.

Using the predicted myopia status from both models, we further evaluated model performance by calculating the following: (1) the predicted and observed myopia prevalence rate overall and by age group; (2) sensitivity, specificity, and their 95% confidence intervals (95% CI) for predicting myopia status in an eye (e.g., eye-level analysis) and in a student (e.g., person-level analysis). In person-level analyses, predicted person-level myopia positive was defined as predicted cycloplegic SER −0.5 D or worse in either eye from the XGBoost model, or myopia positive in either eye from the random forest model. In all eye-level analyses, the inter-eye correlation was accounted for by using generalized estimating equations [24]. Eye-level analysis provides a more precise estimate of machine learning model performance for predicting refractive error, whereas person-level prediction of myopia status is more clinically meaningful and directly relevant for estimating myopia prevalence using machine learning models. These statistical analyses of data from the ML output were performed in SAS v9.4 (SAS Institute Inc., Cary, NC, USA). The study was designed to enroll a total of 600 students which will provide a half width of 0.033 for 95% confidence interval of sensitivity and specificity assuming a myopia prevalence rate of 50% and 90% sensitivity and specificity of the machine learning model.

## 3. Results

The study included 1221 eyes from 614 students. The demographic and ocular characteristics of the students are summarized in Table 1. The mean (SD) age was 11 (1.8) years (range: 8 to 13 years), 45% were female, and 36% wore refractive correction. A dose of 0.5% tropicamide was used in 505 (82.2%) students, and 1% cyclopentolate hydrochloride was used in 109 (17.8%) students. Ocular biometry was performed using the IOLMaster 700 in 207 (33.7%) students and the Optical Biometer SW-9000 in 407 (66.3%) students.

The mean (SD) cycloplegic SER was −0.93 (1.92) D, with 54.5% of eyes having myopia. Mean non-cycloplegic SER was −1.45 (1.79) D, with 67.3% of eyes having non-cycloplegic SER ≤ −0.5 D. UCVA of 20/200 or worse occurred in 5.6% of eyes and UCVA of 20/20 or better occurred in 36.5% of eyes. The mean (SD) for ocular biometric measurements was 24.2 (1.1) mm for AL, 7.90 (0.28) for CR, 3.07 (0.13) for AL/CR ratio, 550 (31) μm for central corneal thickness, 3.31 (0.34) mm for anterior chamber depth, and 16.9 (2.4) mmHg for intraocular pressure. Compared to the internal dataset,^17^ participants in the external validation dataset were older (11.3 years vs. 9.9 years), there was a higher percentage of students wearing refractive correction (36.0% vs. 20.5%), more myopic cycloplegic refractive error (−0.93 D vs. −0.35 D), longer axial length (24.2 vs. 23.6 mm), worse uncorrected visual acuity (40.2% vs. 29.3% with corrected visual acuity worse than 20/40).

### 3.1. XGBoost for Predicting Cycloplegic SER and Myopia Status

**Figure 1:**
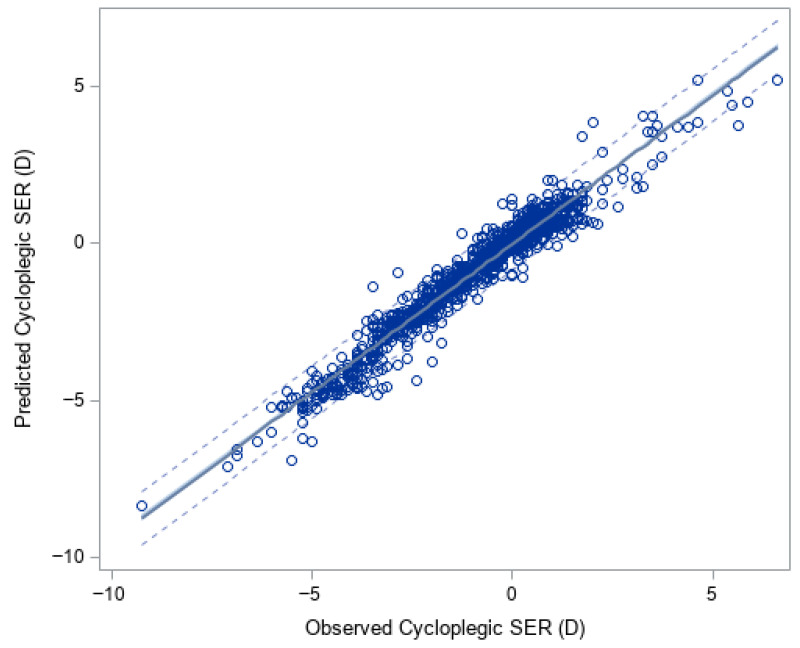
Observed versus predicted spherical equivalent refraction (SER) from XGBoost (R^2^ = 0.97). The solid line is the linear regression line, and dashed lines are 95% confidence limits.

**Figure 2:**
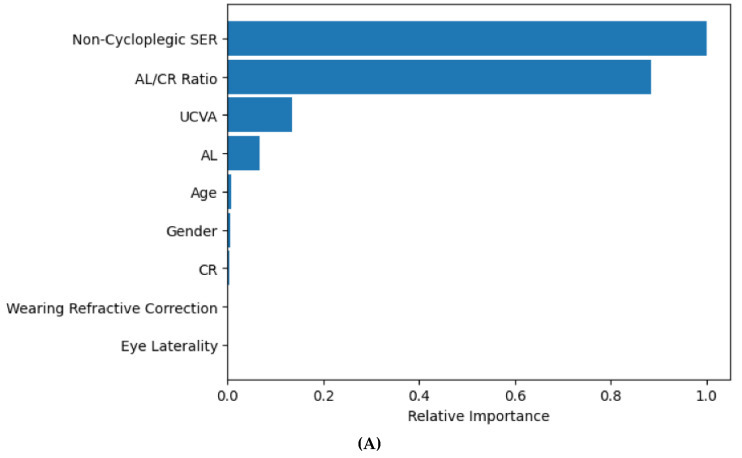

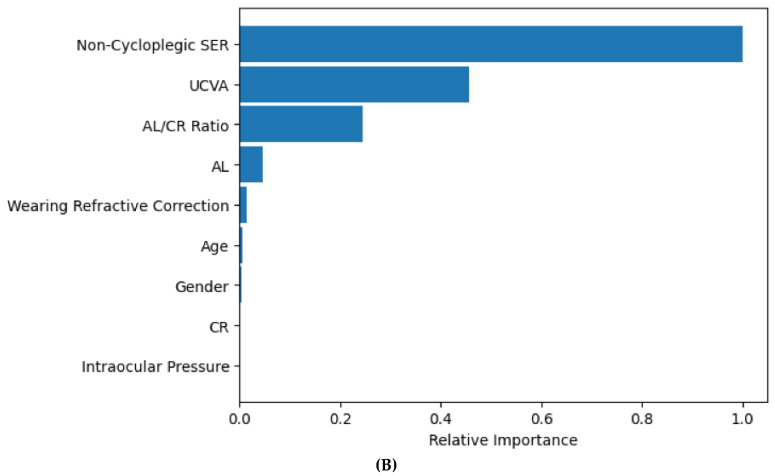
Feature Importance for predicting SER using XGBoost (**A**), and for predicting myopia using random forest (**B**).

The XGBoost model predicted cycloplegic SER very well with R^2^ 0.95 and an MAE (SD) of 0.32 (0.30) D (Table 2, Figure 1). The mean predicted cycloplegic SER was −0.89 (1.86) D, very close to the mean observed cycloplegic SER of −0.93 (1.92) D, with a mean (SD) difference of 0.05 (0.44) D (95% limits of agreement: −0.81 to 0.91 D). When the predicted and observed cycloplegic SER were categorized into six severity levels (≤−6.0 D, >−6.0 to ≤−3.0 D, >−3.0 to ≤−0.5 D, >−0.5 to ≤0.5 D, >0.5 D to ≤3.0 D, >3.0 D), the XGBoost predicted SER showed good agreement with the observed SER with a weighted Kappa of 0.84 (95% CI: 0.82–0.86, Table 3). The most important features for predicting cycloplegic SER by the XGBoost were non-cycloplegic SER, AL/CR ratio, UCVA, and AL (Figure 2A).

Using XGBoost predicted cycloplegic SER ≤−0.5 D as myopia; the XGBoost had a sensitivity of 90.1% (95% CI: 87.3–92.3%) and specificity of 96.8% (95% CI: 94.8–98.0%) for predicting myopia in an eye (i.e., from per-eye analysis), and a sensitivity of 90.7% (95% CI: 87.8–93.6%) and specificity 95.2% (95% CI: 92.4–98.0%; Table 4) for predicting myopia in a student (i.e., from per-person analysis).

### 3.2. XGBoost for Predicting Cycloplegic SER by Age Group and Refractive Error

We further assessed the performance of XGBoost for predicting cycloplegic SER based on age and the magnitude of the cycloplegic SER (Table 2). The model performed consistently well for different age groups ranging from 8 to 13 years, with R^2^ 0.90 (for age 8 years) to 0.96 (for age 13 years), MAE from 0.30 D (for age 10 years) to 0.34 D (for age 8 years). The mean difference between predicted and observed cycloplegic SER ranged from −0.02 D to 0.07 D (Table 2), and there were no statistically significant differences among age groups in both mean difference (*p* = 0.62) and mean absolute difference (*p* = 0.84).

When model performance was evaluated based on the magnitude of the cycloplegic SER, the model also performed well, with a MAE of 0.29 D (for eyes with cycloplegic SER −0.5 to ≤0.5 D) to 0.39 D (for eyes with cycloplegic SER ≤ −3.0 D), and a mean difference between predicted and observed cycloplegic SER of −0.21 D (for eyes with SER > 0.5 D) to 0.14 D (for eyes with SER −3.0 D to ≤−0.50 D; Table 2). There were statistically significant differences among cycloplegic SER groups in both mean differences (*p* < 0.0001) and mean absolute differences (*p* = 0.004, Table 2). We compared the demographic and ocular characteristics between eyes with good XGboost prediction performance (difference between predicted and observed cycloplegic SER < 0.5 D) versus eyes with poor prediction performance (difference ≥ 0.5 D). Eyes with poor prediction were more likely to be female (52.0% vs. 43.2%, *p* = 0.04), have shorter axial length (23.9 vs. 24.2 mm, *p* = 0.0002), lower corneal curvature radius (7.85 vs. 7.91 mm, *p* = 0.02), and lower anterior chamber depth (3.22 vs. 3.33 mm, *p* < 0.0001).

### 3.3. Random Forest Model for Predicting Myopia Status

**Figure 3:**
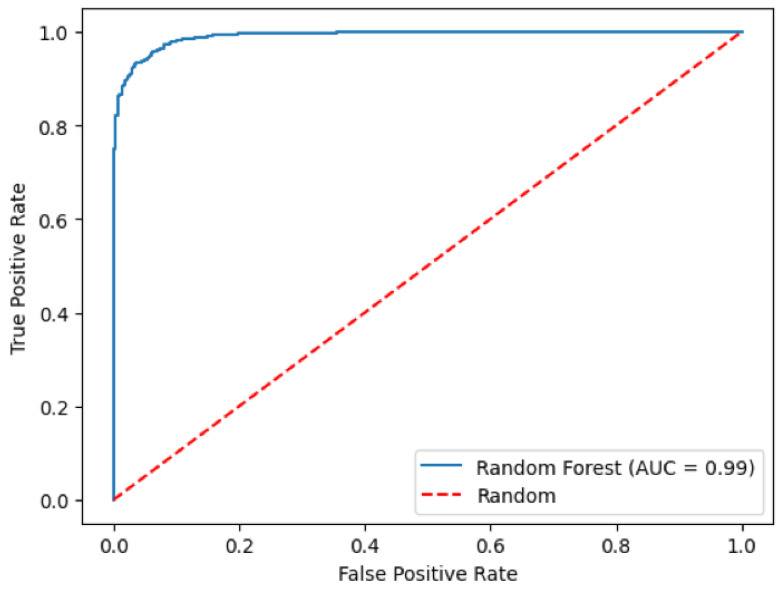
Receiver Operating Characteristic (ROC) Curve for random forest predicting myopia.

The random forest model performed very well in predicting myopia status, with an AUC of 0.991 (95% CI: 0.988, 0.995; Figure 3) and an accuracy of 94.8%. The most important features identified by the random forest model for predicting myopia status were non-cycloplegic SER, UCVA, AL/CR ratio, and AL (Figure 2B). The random forest model had a sensitivity of 93.4% (95% CI: 91.1% to 95.1%) and a specificity of 96.4% (95% CI: 94.4% to 97.7%) for predicting myopia status in an eye, and a sensitivity of 93.3% (95% CI: 90.3% to 95.6%) and a specificity of 94.7% (95% CI: 91.8% to 97.6%) for predicting myopia status in a student. Eyes with incorrectly predicted myopia status by random forest were less likely to be wearing refractive correction (4.7% vs. 37.7%, *p* < 0.0001), less myopic cycloplegic SER (−0.48 D vs. −0.96 D, *p* < 0.0001), less myopic non-cycloplegic SER (−0.96 D vs. −1.47 D, *p* < 0.0001), smaller axial length/corneal curvature radius ratio (3.04 vs. 3.07, *p* = 0.002), and lower percent of uncorrected visual acuity worse than 20/40 (0% vs. 42.4%, *p* < 0.0001).

### 3.4. XGBoost and Random Forest for Predicting Myopia Prevalence Rate

**Figure 4:**
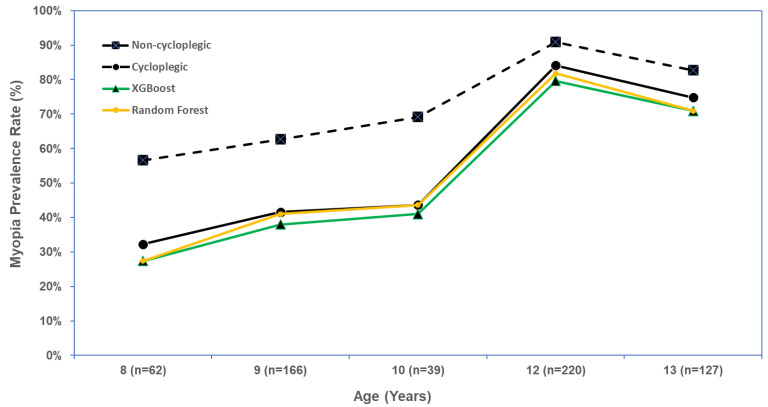
Myopia prevalence rate by age based on the non-cycloplegic, cycloplegic, and machine learning model predictions.

We evaluated XGBoost and random forest for predicting the myopia prevalence rate overall and by age group. Defining myopia-positive as the XGBoost-predicted cycloplegic SER ≤ −0.5 D in either eye, the predicted overall myopia prevalence rate was 58.8% (95% CI: 54.8%–62.7%) which was similar to the observed myopia prevalence rate of 62.9% (95% CI: 59.1%–66.7%). The predicted myopia prevalence rate was similar to the observed rate in each age group, with all differences within 5% (Table 5, Figure 4).

The random forest model predicted an overall myopia prevalence rate of 60.6% (95% CI: 56.6%–64.4%), which was close to the observed myopia prevalence rate of 62.9% (95% CI: 59.1%–66.7%). In each age group, the random forest model predicted the myopia prevalence rate well, with differences between predicted and observed myopia prevalence rates all falling within 5% (Table 5, Figure 4).

Estimating the overall myopia prevalence rate using non-cycloplegic SER ≤ −0.5 D was 76.7% (95% CI: 73.2–80.0%), significantly higher than the observed myopia prevalence rate from cycloplegic SER (62.9%), and the predicted myopia prevalence rate from either model (XGBoost, 58.8%; random forest, 60.6%; Table 5, Figure 4).

### 3.5. Model Prediction Performance Stratified by Cycloplegic Agent and Ocular Biometer

The XGBoost model performed well for predicting cycloplegic SER for eyes receiving both cycloplegic agents: 1.0% cyclopentolate (R^2^ = 0.86, MAE = 0.35 D) and 0.5% tropicamide (R^2^ = 0.95, MAE = 0.32 D). XGBoost also performed similarly well for predicting cycloplegic SER using biometric measures from both biometers: IOLMaster 700 (R^2^ = 0.95, MAE = 0.31 D) and Optical Biometer SW-9000 (R^2^ = 0.94, MAE = 0.33 D; Table 6).

The random forest model performed equally well for predicting myopia status in eyes using a cycloplegic agent of either 1.0% cyclopentolate (AUC = 0.991) or 0.5% tropicamide (AUC = 0.990), or using biometric measures from either IOLMaster 700 (AUC = 0.992), or from Optical Biometer SW-9000 (AUC = 0.991; Table 6).

## 4. Discussion

In this study, we performed an external validation of machine learning models for predicting myopia on a large, independent cohort of students with different cycloplegia and ocular biometry protocols. We specifically validated the two best-performing models that we previously developed and validated in two large cohorts using a standardized protocol for cycloplegia and ocular biometry. In this new cohort, XGBoost continued to demonstrate excellent performance for predicting cycloplegic SER, and random forest accurately predicted myopia status using non-cycloplegic data. This study demonstrates the robustness and generalizability of these models for predicting cycloplegic refractive error and myopia status even when using a non-standardized protocol. The findings from this study support the use of these ML models for determining the presence and severity of myopia in individual students and for population-level estimates of myopia prevalence in large epidemiological studies when measuring cycloplegic SER is not always feasible.

We previously developed and validated several ML models for predicting cycloplegic SER and myopia status based on non-cycloplegic data. In the training and validation datasets, the XGBoost model performed best for predicting cycloplegic SER, and the random forest model performed best for predicting myopia status. Both models yielded high sensitivity and specificity for predicting myopia [18]. Given this, we chose these two models for this external validation study that used different study protocols of cycloplegic refraction (1% cyclopentolate or 0.5% tropicamide as cycloplegic agents) and ocular biometers (Optical Biometer SW-9000 or IOLMaster 700). Despite these differences, the two best-performing models continued to perform very well for the two different cycloplegic agents and ocular biometers. These models also performed very well in all age groups (8 to 13 years). The consistent excellent prediction performances of both models across sub-groups support their use as a tool for determining myopia presence and severity based on measurements obtained under non-cycloplegic conditions.

Our ML models considered ocular biometric measures that can be reliably measured without cycloplegia. Consistent with our previous study, this study found that the non-cycloplegic SER, AL, AL/CR ratio, and UCVA were the top four most important features for predicting cycloplegic SER and myopia status [18]. These measures are well-known to be associated with cycloplegic refractive error and have been used for predicting cycloplegic refractive error in prior studies [8,13,14,16,25]. In this study, we evaluated XGBoost for predicting both cycloplegic refractive error (as a continuous variable) and myopia status (as a binary variable) by defining myopia as predicted cycloplegic SER ≤ −0.5 D. Predicting cycloplegic refractive error can be useful both for detecting myopia and quantifying myopia severity because the predicted cycloplegic SER (a continuous measure) can be later used to define the presence of myopia and to determine the severity of myopia based on the magnitude of the predicted cycloplegic SER. XGBoost performed very well at predicting cycloplegic SER (R^2^ 0.97, MAE 0.32 D) and had high sensitivity and specificity for predicting both myopia and myopia severity level.

Identifying the prevalence or incidence rate of myopia based on non-cycloplegic data could be of interest to large epidemiology studies where cycloplegia is not feasible, and ML models may have great promise in this context. Obtaining cycloplegic data on every child in a population-based study is challenging, in that it requires administration of multiple sets of eye drops, waiting 30 min for complete cycloplegia, and many students may be unwilling to participate. When comparing the ML-model-predicted myopia prevalence rate with the observed myopia rate, we found that the predicted rates from both models were very close to the observed rate. In contrast, the myopia prevalence rate using non-cycloplegic refractive error alone substantially over-estimated the true rate, supporting the use of XGBoost and random forest in future epidemiological studies of myopia. Because refractive error changes with age, we evaluated the performance of XGBoost and random forest for predicting myopia prevalence in each age group and found that both models performed consistently well across all age groups, supporting their predictive value in various age groups.

The participants in this study underwent cycloplegia using two different agents: 1% cyclopentolate and 0.5% tropicamide. 1% cyclopentolate is a well-accepted cycloplegic agent in children, while tropicamide is an alternative agent that potentially has less toxicity and side effects compared to cyclopentolate in children [26]. Previous studies have demonstrated similar cycloplegic effects with these two agents, with mean differences ranging from −0.08 D to 0.54 D, and a pooled mean refractive error difference of 0.21 D (95% CI: 0.08 to 0.35 D) [27]. The similar predictive performance we demonstrated with both cycloplegic agents supports the potential “real world” application of these models.

As compared to traditional regression models, machine learning models more aptly evaluate nonlinear relationships, and thus demonstrate great promise for predicting cycloplegic refractive error based on non-cycloplegic data [17,18,19,20,28,29]. Similarly to our work, Du et al. also evaluated various machine learning algorithms that showed good performance for predicting cycloplegic refractive error with R^2^ 0.889–0.927, MAE 0.372–0.436 D, and for predicting refractive error status with an accuracy of 80.3–81.7% [17]. A recent study by Chen et al. evaluated six machine learning models for predicting cycloplegic refractive error using demographics, non-cycloplegic refractive error, and ocular biometrics. Their model also performed well for predicting cycloplegic SER with high R^2^ (0.920∼0.934), and low MAE (0.385∼0.413 D) [19]. Although these models demonstrated promising potential for predicting cycloplegic refractive error in clinical and epidemiological settings, their models have not been fully validated in an independent sample. It is important to perform independent external model validation in future studies.

While this study validated the excellent performance of the XGBoost model for predicting cycloplegic spherical equivalent refraction (SER), and the random forest model for predicting myopia status using non-cycloplegic data, it has several limitations. These include the enrollment of students from a single city within a narrow age range (8 to 13 years), and the use of only two cycloplegic agents (1% cyclopentolate or 0.5% tropicamide) as well as two different ocular biometers (IOL Master 700 or SW-9000). These factors may limit the generalizability of our findings. Future validation studies involving students from both urban and rural schools, using a broader range of cycloplegic agents and different ocular biometers, would be valuable in expanding the potential applications of these machine learning models in myopia research and clinical care.

## 5. Conclusions

We demonstrated a successful external validation of the XGBoost and random forest ML models for predicting cycloplegic refractive error and myopia using a non-standard cycloplegia and biometry protocol. The consistent excellent performance of these models in heterogenous conditions shows that ML models hold promise in addressing the challenges associated with performing cycloplegia in large epidemiological studies. Further work is needed to validate these models in other contexts.

## Figures and Tables

**Table 1:** Characteristics of study participants.

**Demographic Characteristics**	**(N = 614 Participants)**
Age (Years)	n (%)
8	62 (10.1%)
9	166 (27.0%)
10	39 (6.4%)
12	220 (35.8%)
13	127 (20.7%)
Mean (SD)	11.3 (1.8)
	
Sex: Female (%)	276 (45.0%)
Wearing refractive correction (%)	221 (36.0%)
	
Cycloplegic agent	
0.5% Tropicamide	505 (82.2%)
1% Cyclopentolate Hydrochloride	109 (17.8%)
	
Ocular Biometer	
IOLMaster 700	207 (33.7%)
Optical Biometer SW-9000	407 (66.3%)
	
**Ocular characteristics**	**(N = 1221 eyes)**
Cycloplegic SER (diopter)	Number of eyes (%)
≤−6.0	7 (0.6%)
>−6.0 to ≤−3.0	186 (15.2%)
>−3.0 to ≤−0.5	472 (38.7%)
>−0.5 to ≤0.5	279 (22.9%)
>0.5 to ≤3.0	257 (21.1%)
>3.0	20 (1.6%)
Mean (SD)	−0.93 (1.92)
	
Non-cycloplegic SER (diopter)	
≤−6.0	12 (1.0%)
>−6.0 to ≤−3.0	226 (18.5%)
> −3.0 to ≤−0.5	583 (47.8%)
> −0.5 to ≤0.5	321 (26.3%)
>0.5 to ≤3.0	70 (5.7%)
>3.0	9 (0.7%)
Mean (SD)	−1.45 (1.79)
	
Uncorrected visual acuity	
20/200 or worse	68 (5.6%)
>20/200–20/100	203 (16.6%)
>20/100–20/50	220 (18.0%)
20/40	80 (6.6%)
20/33	103 (8.4%)
20/25	101 (8.3%)
20/20 or better	446 (36.5%)
Intraocular pressure (mmHg): Mean (SD)	16.9 (2.4)
	
**Ocular Biometric Values**	Mean (SD)
Axial length (mm)	24.2 (1.1)
Corneal curvature radius (mm)	7.90 (0.28)
Axial length/corneal curvature radius ratio	3.07 (0.13)
Anterior chamber depth (mm)	3.31 (0.34)
Central corneal thickness (μm)	550 (31)
SER = Spherical equivalent refraction; SD = standard deviation	

**Table 2:** Performance of XGBoost for predicting cycloplegic refractive error overall, by age group, and by refractive error levels.

	Eyes	Observed Mean (SD)	Predicted Mean (SD)	Mean Difference (Predicted–Observed) (95% Confidence Interval) (Predicted–Observed)	Absolute Difference (95% Confidence Interval) (Predicted–Observed)	R^2^
**Overall**	1221	−0.93 (1.92)	−0.89 (1.86)	0.05 (0.02, 0.07)	0.32 (0.31, 0.34)	0.95
						
**By age (Years)**						
8	120	0.16 (1.43)	0.19 (1.28)	0.03 (−0.05, 0.11)	0.34 (0.29, 0.40)	0.90
9	330	−0.14 (1.39)	−0.10 (1.28)	0.04 (−0.01, 0.08)	0.32 (0.29, 0.36)	0.90
10	78	−0.11 (1.52)	−0.13 (1.50)	−0.02 (−0.10, 0.06)	0.30 (0.25, 0.34)	0.94
12	440	−1.61 (1.89)	−1.55 (1.89)	0.06 (0.02, 0.11)	0.32 (0.29, 0.35)	0.95
13	253	−1.57 (2.14)	−1.50 (2.08)	0.07 (0.01, 0.12)	0.33 (0.29, 0.37)	0.96
						
**By cycloplegic spherical equivalent refraction (Diopter)**						
≤−3.0	193	−4.12 (0.94)	−4.03 (1.06)	0.09 (0.02, 0.17)	0.39 (0.34, 0.43)	0.88
>−3.0 to ≤−0.5	472	−1.55 (0.72)	−1.40 (0.81)	0.14 (0.11, 0.18)	0.30 (0.27, 0.32)	0.89
>−0.5 to ≤0.5	279	0.11 (0.30)	0.22 (0.47)	0.11 (0.07, 0.15)	0.29 (0.26, 0.32)	0.61
>0.5	277	1.28 (0.96)	1.07 (0.89)	−0.21 (−0.26, −0.15)	0.37 (0.32, 0.41)	0.88

**Table 3:** The agreement between XGBoost-predicted and observed cycloplegic spherical equivalent levels.

	Observed Cycloplegic Spherical Equivalent (Diopter)						
**Predicted Cycloplegic Spherical Equivalent from XGBoost (Diopter)**	≤−6.0	>−6.0 to ≤−3.0	>−3.0 to ≤−0.5	>−0.5 to ≤0.5	>0.5 to ≤3.0	>3.0	Total
≤−6.0	6 (0.5%)	3 (0.3%)	0	0	0	0	9 (0.7%)
>−6.0 to ≤−3.0	1 (0.1%)	144 (11.8%)	7 (0.6%)	0	0	0	152 (12.5%)
>−3.0 to ≤−0.5	0	39 (3.2%)	399 (32.7%)	18 (1.5%)	0	0	456 (37.4%)
>−0.5 to ≤0.5	0	0	66 (5.4%)	181 (14.8%)	38 (3.1%)	0	285 (23.3%)
>0.5 to ≤3.0	0	0	0	80 (6.6%)	217 (17.8%)	5 (0.4%)	302 (24.7%)
>3.0	0	0	0	0	2 (0.2%)	15 (1.2%)	17 (1.4%)
Total	7 (0.6%)	186 (15.2%)	472 (38.7%)	279 (22.9%)	257 (21.1%)	20 (1.6%)	1221 (100%)
Percent agreement = 78.8%, weight Kappa = 0.84 (0.82, 0.86)							

**Table 4:** Sensitivity and specificity for predicting myopia. Myopia based on predicted cycloplegic SER (XGBoost model) or myopia status yes/no (random forest model).

	Per-Eye Analysis		Per-Person Analysis	
Model	Sensitivity (95% CI)	Specificity (95% CI)	Sensitivity (95% CI)	Specificity (95% CI)
**Using predicted cycloplegic SER ≤ −0.5 D from XGBoost**	90.1% (87.3%, 92.3%)	96.8% (94.8%, 98.0%)	90.7% (87.8%, 93.6%)	95.2% (92.4%, 98.0%)
**Using predicted myopia yes/no from Random Forest**	93.4% (91.1%, 95.1%)	96.4% (94.4%, 97.7%)	93.3% (90.3%, 95.6%)	94.7% (91.8%, 97.6%)

SER = Spherical equivalent refraction; 95% CI = 95% confidence interval.

**Table 5:** The observed myopia prevalence rate using non-cycloplegic and cycloplegic refractive error, predicted cycloplegic refractive error from the XGBoost, and predicted myopia status from random forest. Data are presented as number (percent) of students.

		Myopia Rate (95% Confidence Interval)			
	Number of Students	Observed (Using Non-Cycloplegic SER ≤ −0.5 D in Either Eye)	Observed (Using Cycloplegic SER ≤ −0.5 D in Either Eye)	XGBoost (Using Predicted Cycloplegic SER ≤ −0.5 D in Either Eye)	Random Forest (Using the Predicted Presence of Myopia in Either Eye)
**Overall**	614	471 (76.7%) (73.4–80.1%)	386 (62.9%) (59.0–66.7%)	361 (58.8%) (54.9–62.7%)	372 (60.6%) (56.7–64.5%)
**By age (Years)**					
8	62	35 (56.5%) (44.1–68.8%)	20 (32.3%) (20.6–43.9%)	17 (27.4%) (16.3–40.2%)	17 (27.4%) (16.3–38.5%)
9	166	104 (62.7%) (55.3–70.0%)	69 (41.6%) (34.1–49.1%)	63 (38.0%) (30.6–45.3%)	68 (41.0%) (33.5–48.4%)
10	39	27 (69.2%) (54.8–83.7%)	17 (43.6%) (28.0–59.2%)	16 (41.0%) (25.6–56.5%)	17 (43.6%) (28.0–59.2%)
12	220	200 (90.9%) (87.1–94.7%)	185 (84.1%) (79.3–88.9%)	175 (79.6%) (73.6–84.7%)	180 (81.8%) (76.7–86.9%)
13	127	105 (82.7%) (76.1–89.3%)	95 (74.8%) (67.3–82.4%)	90 (70.9%) (63.0–78.8%)	90 (70.9%) (63.0–78.8%)

**Table 6:** Prediction performance of XGBoost and random forest by type of cycloplegic agent and ocular biometer.

				XGBoost for Predicting Cycloplegic SER			Random Forest for Predicting Myopia Status
Subgroups	Number of eyes	Observed Cycloplegic SER (SD)	Predicted SER (SD)	Mean Difference (Predicted– Observed) (SD)	Mean Absolute Difference (SD)	R^2^	AUC (95% CI)
**Overall**	1221	−0.93 (1.92)	−0.89 (1.86)	0.05 (0.44)	0.32 (0.30)	0.95	0.991 (0.988, 0.995)
							
**By Cycloplegic agent**							
Cyclopentolate	212	0.22 (1.29)	0.22 (1.16)	0.004 (0.49)	0.35 (0.34)	0.86	0.991 (0.983, 0.999)
Tropicamide	1009	−1.18 (1.94)	−1.12 (1.90)	0.06 (0.43)	0.32 (0.29)	0.95	0.990 (0.987, 0.994)
**By Ocular Biometer**							
IOLMaster 700	407	−0.32 (1.70)	−0.30 (1.61)	0.02 (0.43)	0.31 (0.29)	0.95	0.992 (0.986, 0.997)
Optical Biometer SW-9000	814	−1.24 (1.95)	−1.18 (1.91)	0.06 (0.45)	0.33 (0.31)	0.94	0.991 (0.986, 0.995)

SER = Spherical equivalent refraction; AUC = area under the receiver operating characteristic (ROC) curve; SD = standard deviation; 95% CI = 95% confidence interval.

## Data Availability

The original contributions presented in this study are included in the article. Further inquiries can be directed to the corresponding author.

## References

[1] Sankaridurg P., Tahhan N., Kandel H., Naduvilath T., Zou H., Frick K.D., Marmamula S., Friedman D.S., Lamoureux E., Keeffe J. (2021). IMI Impact of Myopia. Investig. Ophthalmol. Vis. Sci..

[2] Flitcroft D.I., He M., Jonas J.B., Jong M., Naidoo K., Ohno-Matsui K., Rahi J., Resnikoff S., Vitale S., Yannuzzi L. (2019). IMI—Defining and Classifying Myopia: A Proposed Set of Standards for Clinical and Epidemiologic Studies. Investig. Ophthalmol. Vis. Sci..

[3] Koh V., Tan C., Nah G., Zhao P., Yang A., Lin S.T., Wong T.Y., Saw S.M., Chia A. (2014). Correlation of structural and electrophysiological changes in the retina of young high myopes. Ophthalmic Physiol. Opt..

[4] Wu H.M., Seet B., Yap E.P., Saw S.M., Lim T.H., Chia K.S. (2001). Does education explain ethnic differences in myopia prevalence? A population-based study of young adult males in Singapore. Optom. Vis. Sci..

[5] Yotsukura E., Torii H., Inokuchi M., Tokumura M., Uchino M., Nakamura K., Hyodo M., Mori K., Jiang X., Ikeda S.I. (2019). Current Prevalence of Myopia and Association of Myopia with Environmental Factors Among Schoolchildren in Japan. JAMA Ophthalmol..

[6] Yang Y., Liao H., Zhao L., Wang X., Yang X., Ding X., Li X., Jiang Z., Zhang X., Zhang Q. (2024). Green Space Morphology and School Myopia in China. JAMA Ophthalmol..

[7] Wilson S., Ctori I., Shah R., Suttle C., Conway M.L. (2022). Systematic review and meta-analysis on the agreement of non-cycloplegic and cycloplegic refraction in children. Ophthalmic Physiol. Opt..

[8] Gu F., Gao H.M., Zheng X., Gu L., Huang J.Y., Meng J., Li J.J., Gao L., Wang J.Y., Zhang R.H. (2022). Effect of Cycloplegia on Refractive Error Measure in Chinese School Students. Ophthal. Epidemiol..

[9] Foo V.H., Verkicharla P.K., Ikram M.K., Chua S.Y., Cai S., Tan C.S., Chong Y.S., Kwek K., Gluckman P., Wong T.Y. (2016). Axial Length/Corneal Radius of Curvature Ratio and Myopia in 3-Year-Old Children. Transl. Vis. Sci. Technol..

[10] He X., Zou H., Lu L., Zhao R., Zhao H., Li Q., Zhu J. (2015). Axial length/corneal radius ratio: Association with refractive state and role on myopia detection combined with visual acuity in Chinese schoolchildren. PLoS ONE.

[11] Ip J.M., Huynh S.C., Kifley A., Rose K.A., Morgan I.G., Varma R., Mitchell P. (2007). Variation of the contribution from axial length and other oculometric parameters to refraction by age and ethnicity. Investig. Ophthalmol. Vis. Sci..

[12] Kimura S., Hasebe S., Miyata M., Hamasaki I., Ohtsuki H. (2007). Axial length measurement using partial coherence interferometry in myopic children: Repeatability of the measurement and comparison with refractive components. Jpn. J. Ophthalmol..

[13] Magome K., Morishige N., Ueno A., Matsui T.A., Uchio E. (2021). Prediction of cycloplegic refraction for noninvasive screening of children for refractive error. PLoS ONE.

[14] Ojaimi E., Rose K.A., Morgan I.G., Smith W., Martin F.J., Kifley A., Robaei D., Mitchell P. (2005). Distribution of ocular biometric parameters and refraction in a population-based study of Australian children. Investig. Ophthalmol. Vis. Sci..

[15] Sankaridurg P., He X., Naduvilath T., Lv M., Ho A., Smith E., Erickson P., Zhu J., Zou H., Xu X. (2017). Comparison of noncycloplegic and cycloplegic autorefraction in categorizing refractive error data in children. Acta Ophthalmol..

[16] Wang J., Wang X., Gao H.M., Zhang H., Yang Y., Gu F., Zheng X., Gu L., Huang J., Meng J. (2022). Prediction for Cycloplegic Refractive Error in Chinese School Students: Model Development and Validation. Transl. Vis. Sci. Technol..

[17] Du B., Wang Q., Luo Y., Jin N., Rong H., Wang X., Nian H., Guo L., Liang M., Wei R. (2023). Prediction of spherical equivalent difference before and after cycloplegia in school-age children with machine learning algorithms. Front. Public Health.

[18] Ying B., Chandra R.S., Wang J., Cui H., Oatts J. (2024). Machine Learning Models for Predicting Cycloplegic Refractive Error and Myopia Status Based on Noncycloplegic Data in Chinese Students. Transl. Vis. Sci. Technol..

[19] Chen B., Tian L., Tian F., Yang Q., Ruan Y., Li Y., Cao M., Wu C., Yang M., Xu S. (2025). Machine learning-driven prediction of cycloplegic refractive error in Chinese children. Front. Cell Dev. Biol..

[20] Feng Q., Wu X., Liu Q., Xiao Y., Zhang X., Chen Y. (2025). Interpretable machine learning models for predicting childhood myopia from school-based screening data. Sci. Rep..

[21] Breiman L. (2001). Random Forest. Mach. Learn..

[22] Chen T., Guestrin C. XGBoost: A Scalable Tree Boosting System. Proceedings of the 22nd ACM SIGKDD International Conference on Knowledge Discovery and Data Mining.

[23] Pedregosa F., Varoquaux G., Gramfort A., Michel V., Thirion B., Grisel O., Blondel M., Prettenhofer P., Weiss R., Dubourg V. (2011). Scikit-learn: Machine Learning in Python. J. Mach. Learn. Res..

[24] Ying G.-S., Maguire M.G., Glynn R.J., Rosner B. (2020). Calculating Sensitivity, Specificity, and Predictive Values for Correlated Eye Data. Investig. Ophthalmol. Vis. Sci..

[25] Zhao E., Wang X., Zhang H., Zhao E., Wang J., Yang Y., Gu F., Gu L., Huang J., Zhang R. (2022). Ocular biometrics and uncorrected visual acuity for detecting myopia in Chinese school students. Sci. Rep..

[26] Major E., Dutson T., Moshirfar M. (2020). Cycloplegia in Children: An Optometrist’s Perspective. Clin. Optom..

[27] Yazdani N., Sadeghi R., Momeni-Moghaddam H., Zarifmahmoudi L., Ehsaei A. (2018). Comparison of cyclopentolate versus tropicamide cycloplegia: A systematic review and meta-analysis. J. Optom..

[28] Barraza-Bernal M.J., Ohlendorf A., Sanz Diez P., Feng X., Yang L.H., Lu M.X., Wahl S., Kratzer T. (2023). Prediction of refractive error and its progression: A machine learning-based algorithm. BMJ Open Ophthalmol..

[29] Jiang Y., Shen Y., Wang L., Chen X., Tang J., Liu L., Ma T., Ju L., Chen Y., Ge Z. (2024). Effect of vault on predicting postoperative refractive error for posterior chamber phakic intraocular lens based on a machine learning model. J. Cataract. Refract. Surg..

